# Evaluation of the efficacy, prognosis and safety of dexamethasone in the treatment of different types of non-puerperal mastitis: A retrospective study

**DOI:** 10.1371/journal.pone.0325739

**Published:** 2025-06-11

**Authors:** Na Wang, Gang Hu, Lihua Xu, Lili Gong, Wanju Wang

**Affiliations:** Department of Breast, Wuhan Children’s Hospital (Wuhan Maternal and Child Healthcare Hospital), Tongji Medical College, Huazhong University of Science & Technology, Wuhan, China.; Beni Suef University Faculty of Veterinary Medicine, EGYPT

## Abstract

**Objective:**

To analyze the efficacy and safety of dexamethasone in the treatment of non-puerperal mastitis (NPM), providing a new idea for the treatment of NPM.

**Methods:**

From August 1, 2017 to August 30, 2024, case data were collected from 552 patients with NPM. After grouping according to different treatment options**, the** SPSS statistical software was used for retrospective analysis of the collected data.

**Results:**

The number of days of drug treatment before operation in group B was less than other groups (p < 0.001). The group B had the most significant relief of pain symptoms and shorter time to complete relief of pain than other groups (p < 0.001). There were statistically significant differences between the 5 groups in the time required for pain to disappear and the time required for the volume to be reduced by half after treatment (p < 0.05).The overall efficacy evaluation had the highest effective rate in Group B (100%) and the lowest in Group D (2.10%), and the difference was statistically significant (p < 0.001).No side effects such as abnormalities in liver and kidney functions, water-electrolyte disorders, or peptic ulcers were observed in the five groups during drug treatment. There was no statistically significant difference in the occurrence of side effects such as rash, diarrhoea and hyperglycaemia among patients in the five groups (p > 0.05).The side effects of nausea (vomiting) and skin pigmentation in group E were higher than other groups(p < 0.001). In terms of weight gain (full moon face), nervous excitability (insomnia) and menstrual disorders, group B was lower than other groups (except group D without hormone therapy)(p < 0.001). In terms of postoperative recurrence after ipsilateral breast surgery, the recurrence rates of patients in group B were lower than those of the other four groups, and group D had the highest recurrence rate (8.30%), with a statistically significant difference (p < 0.001). Satisfaction survey found that group B had the highest satisfaction rate other groups (p < 0.05). The number of days required for the volume to be reduced by half after treatment was the most influential factor in the satisfaction survey. At the same time, we found that the obvious effect and recovery rate of GLM group was higher than that of PCM group, and the difference was statistically significant (p < 0.05).

**Conclusion:**

Dexamethasone combined with levofloxacin/Metronidazole in the treatment of NPM has many advantages: first of all, it can significantly relieve the pain symptoms caused by the disease and effectively reduce the size of the lesion. Meanwhile, for the patients who plan to undergo surgery, the number of days of preoperative drug treatment can be reduced, and the overall effective rate is the highest. Secondly, the short-term application of drugs to treat side effects less, high safety; in the meantime, the risk of recurrence of the ipsilateral breast was less and the satisfaction of the patients was higher. The overall significant efficiency and recovery rate of GLM patients were higher than those of PCM patients.

## Introduction

NPM is a group of breast inflammatory diseases of unknown etiology occurring in non-lactating women. The main clinical manifestations of NPM are breast mass and breast abscess, which may form fistulas, sinuses or ulcers in the later stage. The natural course of NPM is approximately 9–12 months, which is characterized by high recurrence rate and long course of the disease [1]. The prevalence of NPM is currently increasing year by year [2], accounting for 0.3–1.9% of all breast diseases worldwide. Approximately 65.5% of cases occur in the first five years after delivery [3], mainly in women aged 30–39 years [4].There are two types of NPM, One is Plasma Cell Mastitis (PCM) [5], including mammary duct ectasia (MDE) or periductal mastitis (PDM). PDM refers to an inflammatory state of the subareolar duct [6]. Zuska disease, a specific manifestation of MDE/PDM, is defined as a milk duct fistula with subareolar abscess and fistula. The other is granulomatous lobular mastitis (GLM) [7]. The diagnosis and treatment of NPM is currently tricky, and the impact of NPM on women is multi-faceted. In terms of diagnosis, the imaging manifestations of NPM are very similar to breast cancer, which can easily lead to misdiagnosis [8]. In the area of treatment, most doctors individualize treatment based on previous treatment experience. Some patients require multiple surgeries, which can easily lead to complications such as scar formation, breast deformation and even difficulty in breastfeeding in the later period. Some patients use glucocorticoids or immunosuppressants for a long time due to their condition, which may cause complications such as gastrointestinal reactions, liver function abnormalities or osteoporosis [9]. If the patient is not diagnosed or treated in a timely manner, some accompanying symptoms may also develop, such as low-grade fever, erythema nodosum of the lower extremities, or arthritis [10]. The impact of NPM on women also includes the fact that some patients have a long course of the disease and their symptoms have not improved significantly, they may have developed psychological disorders such as anxiety or depression [11]. If the disease progresses, persists for a long time, recurs, or spreads to the whole breast, the patient may lose her affected breast, which will cause irreversible damage to the patient. Consequently, researchers are actively exploring and studying the causes of NPM as well as effective treatment options to improve the cure rate of NPM, shorten the duration of the disease, minimize postoperative recurrences, and improve the quality of life of patients with NPM by ensuring that the shape and function of the breasts are not compromised while undergoing treatment.

With regards this, Chinese scholars formulated clinical practice guidelines for NPM Diagnosis and treatment (2021 edition) [1] in 2021. Possible risk factors associated with this disease have been found in clinical practice [12,13], including hormone level, autoimmune reaction, bacterial infection, etc. Relevant studies have also found that possible risk factors for NPM include abnormal lipid metabolism [14], but there is no clear cause and standard treatment. In the daily diagnosis and treatment of NPM in the breast department of our hospital, we commonly use intravenous dexamethasone to treat NPM, and the clinical findings have achieved good therapeutic effect. But there is no consensus on dexamethasone in the treatment of NPM, In addition, dexamethasone is only suitable for short-term use, because long-term use of dexamethasone can lead to iatrogenic adrenal insufficiency [15], so its safety and effectiveness need to be further studied and evaluated.In order to further study the efficacy and safety of dexamethasone in the treatment of NPM, this retrospective study was designed to provide a new clinical reference for the treatment of NPM, further optimize the treatment plan of NPM, and provide a clinical practical basis for the standardized treatment of NPM.

## 1 Data and methods

### 1.1 Study design and setting

**Fig 1:**
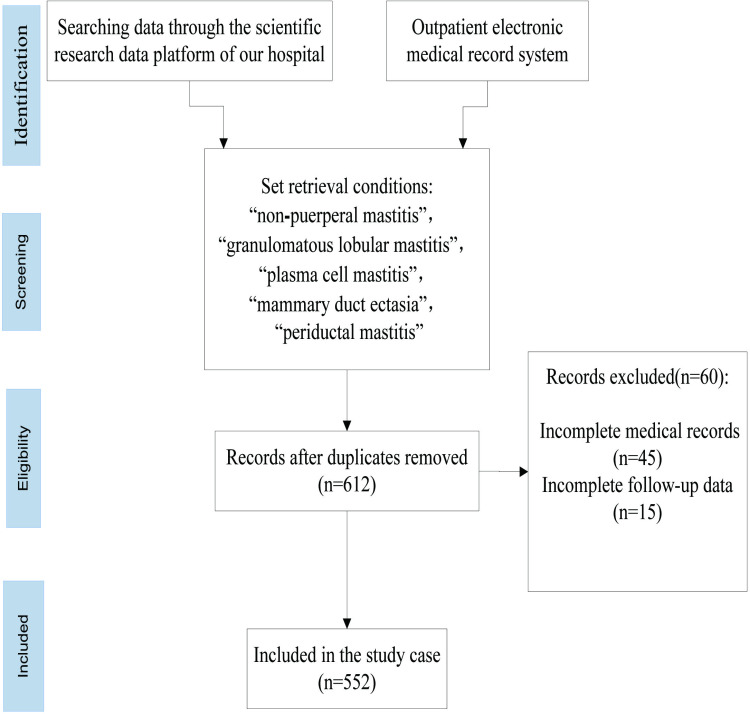
Flow chart of medical record screening.

This retrospective study was approved by the ethics committee of our hospital. The relevant medical records were from our hospital’s scientific research data platform and outpatient electronic medical record system. We accessed them on September 1, 2024. Setting the search conditions in advance, we searched the scientific research data platform of our hospital. The search conditions were “non⁃puerperal mastitis”, “granulomatous lobular mastitis”, “plasma cell mastitis”, “mammary duct ectasia”, or “periductal mastitis”. Patients with NPM admitted to our hospital from August 1, 2017 to August 30, 2024 were screened according to the inclusion and exclusion criteria. The final number of screened medical records was 449 cases that were used as the study population in the experimental group. The outpatient electronic medical record system of our hospital was searched meanwhile, and patients treated with oral prednisone in the outpatient department were selected as the control group, and 103 medical records were finally selected as the control group (Fig 1). According to the retrieved medical records, clinical relevant medical records of the enrolled patients were collected, including medical records before and after drug treatment, follow-up data after discharge, and possible adverse reactions during treatment. Patients were grouped according to different treatment regimens and pathological diagnosis and their medical records were statistically analyzed. This study was approved by the Ethics Committee of our hospital (Ethical examination and approval number: 2024R092-E01). Informed written consent was obtained from all participants for this study to have data from their medical records used in research.

### 1.2 Inclusion criteria and exclusion criteria

The inclusion criteria were:(1) Age ≥ 18 years old; Non-lactating female patients with mastitis who visited and were hospitalized in our hospital; No previous history of NPM; (2) NPM (including PCM, GLM, MDE, etc.) was confirmed by pathology, and the clinical data were complete. Exclusion criteria: (1) lactation mastitis or lactation mammary abscess; (2) Patients with other autoimmune conditions;(3)the pathological diagnosis was breast cancer;(4)medical records are missing.

### 1.3 Grouping

#### (1) Cross-sectional comparison.

Patients were grouped according to their treatment regimen. Group A: dexamethasone alone treatment group. Group B: dexamethasone treatment combined with metronidazole/ levofloxacin treatment group. Group C: dexamethasone treatment combined with cephalosporin antibiotic treatment group. Group D: antibiotic treatment alone group. Outpatient screening of patients treated with oral prednisone served as a control group (group E). Regarding the dose selection of dexamethasone, there is no relevant study on the relationship between the dose of dexamethasone and the treatment of NPM, but studies have found that higher doses of dexamethasone (≥16 mg/day) do not improve the efficacy and rather increase the incidence of adverse events compared with low doses of dexamethasone (8 mg/day) [16], e.g., high blood sugar, etc [17]. Dexamethasone 10 mg/day is a common clinical dose choice, so we chose 10 mg/day of dexamethasone as the therapeutic dose for patients with NPM during routine clinical practice. (2) Longitudinal comparison: The treatment group containing dexamethasone (A + B + C) was divided into GLM group and PCM group according to pathological classification.

### 1.4 Observation indicators

#### 1.4.1 Basic patient information.

They included age, duration of disease, main symptoms, number of births, age at menarche, ever breastfeeding or not and duration of breastfeeding, inverted nipple or not, nipple overflow, body mass index (BMI)(BMI=Weight(kg)/Height(m)*Height(m)), etc.

#### 1.4.2 Main observation indicators.

(1) Curative effect indicators: the efficacy was evaluated by monitoring the changes of pain score and lesion volume before and after treatment. The primary efficacy outcome measures: the time required for the volume to be reduced by half (days), overall efficacy evaluation, pain score and time taken for pain to disappear (days). The secondary efficacy outcome measures: the number of days of preoperative medication. Specifically, as follows:①Pain score: Pre-treatment pain scores, post-treatment pain scores on day 1 as well as day 2, and time taken for pain to disappear (days) were observed, and pain scores were recorded using the numerical rating scale (NRS) (Table 1). ②Monitoring of changes in lesion volume and nipple discharge: the number of days required to halve the volume after treatment was monitored (because some patients had the surgery before the lesion had completely retreated, we chose to monitor the number of days required to halve the volume after treatment to evaluate the therapeutic effect).In the treatment of NPM, we observed that inpatients basically underwent further surgical treatment after one week of treatment, so we chose to evaluate the overall drug efficacy of the patients included in the study after one week of drug treatment. Color ultrasound combined with a physical examination was adopted to evaluate the changes in lesion volume, and we set the evaluation criteria as follows: ① recovery: Color ultrasound indicated that the lump disappeared, the pain disappeared, and the discharge disappeared; ② obvious curative effect: the mass shrinked by more than 1/2 volume, the pain disappears, and the nipple discharge disappears. ③ Effective: the mass shrank less than 1/2 volume, the pain was significantly reduced, and the volume of nipple discharge was reduced. The capillary dilation and congestion and the white filamentous or fibrous network structure in the lumen were all significantly reduced under the ductoscopy. ④invalid: there was no significant reduction in the mass, pain reduction or no change, no change in nipple discharge, local lesions tended to suppuration or fistula formation, and no improvement in the lesions under ductoscopy. Overall effective rate = recovery rate + significant effective rate + effective rate.

**Table 1:** Numerical Rating Scale(NRS).

Project	score
analgesia	0
Slight pain, bearable	1
	2
	3
The pain affects sleep and is bearable	4
	5
	6
Intense pain, unbearable pain, affecting appetite and sleep	7
	8
	9
	10

(2) Safety evaluation: we set up drug-related safety evaluation indicators. The main concern was to monitor the occurrence of adverse reactions, including nausea, vomiting, rash, diarrhoea, peptic ulcers (perforation or bleeding), hypertension, hyperglycemia, weight gain (full-moon face), menstrual disorders, increased skin pigmentation, nervousness (insomnia), or electrolytic disorders, etc. And the changes of liver and kidney function were also monitored.
(3) Prognosis measures: the follow-up data of patients included in this retrospective study were retrieved for 1 year after discharge from the hospital and for 1 year for outpatients, and telephone follow-ups were taken to supplement the complete follow-up data for patients with missing data. NPM relapse as well as new occurrence within one year after treatment was used as a prognostic indicator, and they were defined in the current study. NPM relapse definition: patients with pain, redness, swelling and ulceration of the affected breast within one year after recovery and pathologically confirmed NPM were defined as NPM relapse. NPM new occurrence definition: after recovery, new lesions were found in the contralateral breast, and pathological diagnosis was confirmed as NPM.
(4) Satisfaction: satisfaction survey was conducted on the effectiveness of treatment during this treatment cycle, and it was included in the evaluation indicators of this study.

### 1.5 Statistical methods

SPSS27 statistical software was used for statistical analysis of the data in this retrospective study, and Shapiro-Wilk test and histogram were used to test whether the data were consistent with normal distribution. For measures that conformed to normal distribution were expressed as mean±standard deviation, and one way analysis of variance was used for comparisons among groups; the Kruskal-Wallis rank sum test was adopted to measure that did not fit the normal distribution expressed as Median (M) (Inter-quartile Range (IQR)).Count data were expressed as the number of cases (percentage) [n (%)], and comparisons among groups were made using the Chi-square test (χ^2^) or Fisher’s exact test. Rank data were represented by the number of cases (percentage)[n(%)], and comparison between groups was performed by Kruskal-Wallis rank sum test (H test).Independent risk factors affecting NPM satisfaction were analysed using a logistic regression model, and risk factors from the logistic regression analysis were analysed by drawing a forest plot using STATA 15 statistical software. p < 0.05 was considered to be statistically significant.

## 2 Results

### 2.1 Based on the inclusion as well as exclusion criteria

**Fig 2:**
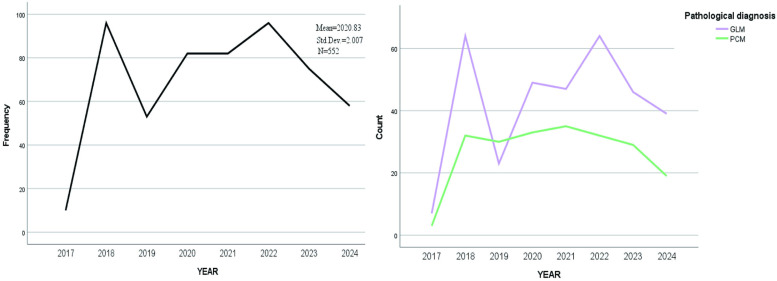
The characteristics of disease year distribution and age distribution.

**Fig 3:**
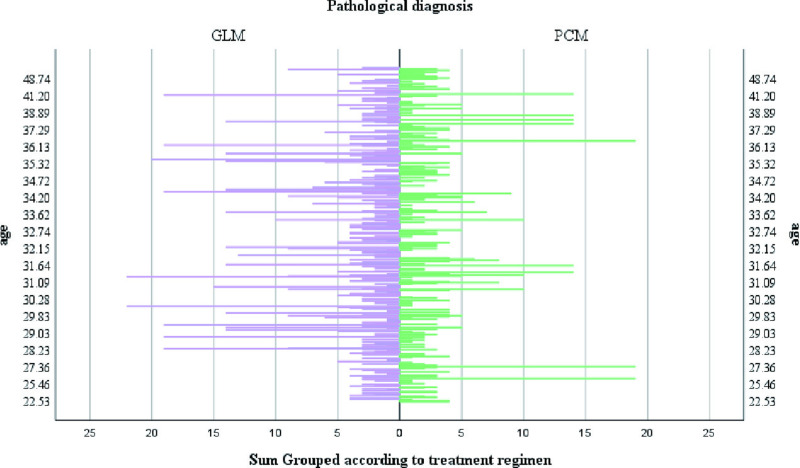
Age distribution characteristics of different pathological types.

**Fig 4:**
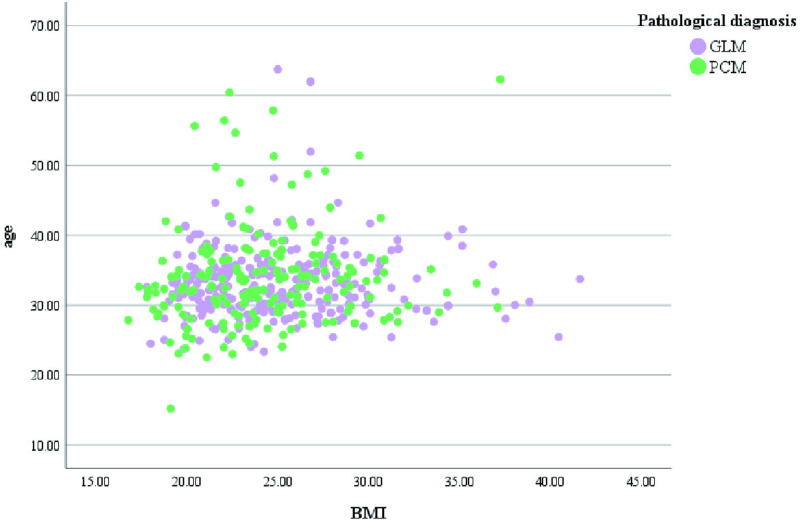
BMI characteristics of different pathological types.

**Fig 5:**
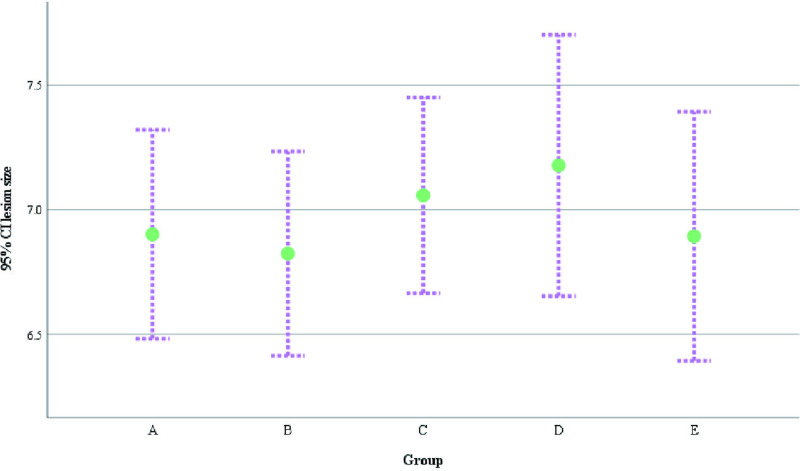
Lesion size (cm).

A total of 552 patients was included in this retrospective study with age ranging from 22.53–63.75 years and median age of 32.47 years. Characteristics of NPM distribution year, age distribution and BMI value in our department: from August 2017 to August 2024, the incidence of NPM had two small peaks in 2018 and 2022 (Fig 2). The age of onset was mainly between 30 and 40 years old (Fig 3). BMI values of patients with different pathological types were mainly concentrated in 20–30 Kg/m^2^ (Fig 4). The size of the lesions on the affected breast ranged from 1.3–14.0 cm, and the median size was 6.96 cm. There were small differences in lesion size changes among the groups (Fig 5).

### 2.2 Statistical analysis of pre-treatment related data of NPM patients included in the study (Table 2)

**Fig 6:**
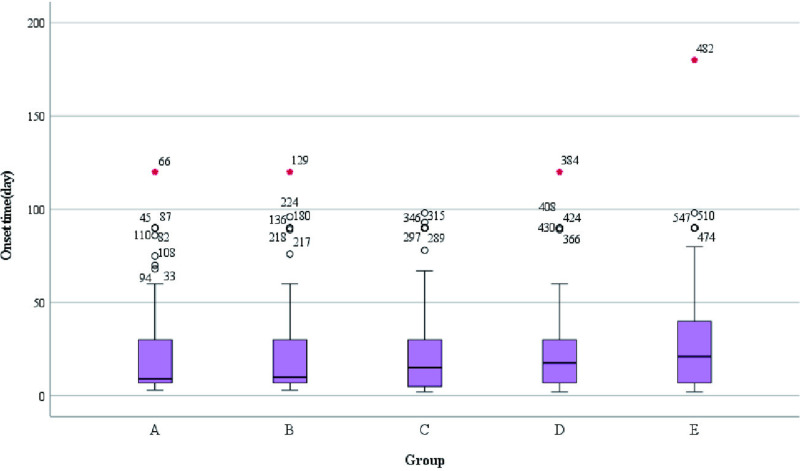
Onset time (day).

There was no statistically significant difference in age, BMI, Childbearing age, age at menarche, total number of pregnancies, duration of breastfeeding, the number of births, location of the affected breast, and type of pathological diagnosis among the five groups (p > 0.05).A box plot was produced for non-normally distributed measures, and it was found that the time to onset of illness was significantly greater in Group E patients than in the other four groups (Fig 6) and the difference in the time to onset of illness among the five groups was statistically significant (H = 12.849, p = 0.012).Statistical analysis of the onset time of patients in groups A, B, C and D was conducted again, and it was found that there was no statistical significance in the onset time of patients in the four groups (H = 1.242, p = 0.743).We looked for reasons for this quadrant through outpatient records as well as by telephone follow-up, and we found that the majority of patients with a long course of disease took outpatient oral medication, specifically because of reasons related to patient non-compliance with the treatment or the choice of treatment regimen of the first physician.

**Table 2:** Initial pre-treatment data grouped according to treatment protocol(mean± standard deviation)/n(%)/(M(IQR)).

Project/Group	A	B	C	D	E	F/H/χ2	p
case	119	109	125	96	103		
age(years),M(IQR)	32.64(6.44)	32.65(5.72)	32.42(6.08)	32.04(5.33)	32.39(5.84)	0.758	0.944
lesion size(cm),M(IQR)	6.50(2.60)	6.70(3.00)	7.00(2.80)	7.00(3.20)	6.70(2.50)	2.109	0.716
BMI(kg/m²),M(IQR)	23.92(5.95)	24.22(4.26)	23.74(5.01)	23.77(6.20)	24.72(5.49)	0.787	0.940
Onset time(day),M(IQR)	9.00(23)	10.00(23)	15.00(25)	17.50(23)	21.00(33)	12.849	0.012
Onset time(day),M(IQR)	9.00(23)	10.00(23)	15.00(25)	17.50(23)	*	1.242	0.743
Childbearing age(years)	26.03 ± 2.91	25.27 ± 2.65	25.68 ± 3.049	25.12 ± 2.90	25.30 ± 2.67	1.850	0.117
total number of pregnancies,M(IQR)	1.00(1)	1.00(1)	1.00(1)	1.00(1)	2.00(1)	7.677	0.104
the number of births,M(IQR)	1.00(1)	1.00(1)	1.00(1)	1.00(1)	1.00(1)	5.688	0.224
location of the affected breast,n(%)						9.011	0.341
Left breast	64.00(53.78)	56.00(51.38)	69.00(55.20)	55.00(57.29)	62(60.19)		
Right breast	54.00(45.38)	48.00(44.04)	53.00(42.40)	40.00(41.67)	41(39.81)		
Bilateral breast	1.00(0.84)	5.00(4.58)	3.00(2.40)	1.00(1.04)	0.00(0.00)		
age at menarche(years),M(IQR)	13.00(1)	13.00(2)	13.00(2)	12.00(1)	12.00(1)	9.308	0.054
duration of breastfeeding(months),M(IQR)	7.00(2)	7.00(2)	7.00(3)	7.00(2)	7.00(1)	5.149	0.272
pathological diagnosis,n(%)						4.899	0.299
GLM	64.00(53.78)	68.00(62.39)	84.00(67.20)	58.00(60.42)	65.00(63.11)		
PCM	55.00(46.22)	41.00(37.61)	41.00(32.80)	38.00(39.58)	38.00(36.89)		

### 2.3 Evaluation of drug therapeutic efficacy of NPM patients included in the study (Table 3)

**Fig 7:**
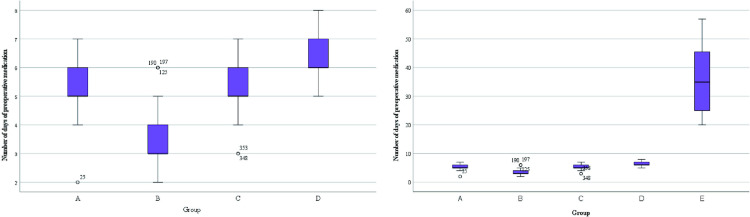
Number of days of preoperative medication.

**Fig 8:**
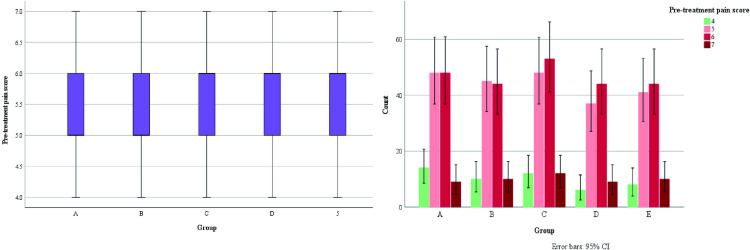
Pre-treatment pain score.

**Fig 9:**
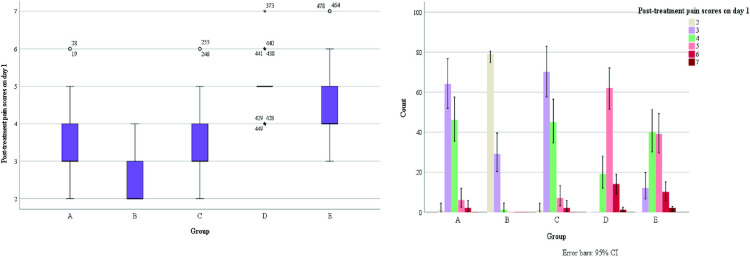
Post-treatment pain scores on day 1.

**Fig 10:**
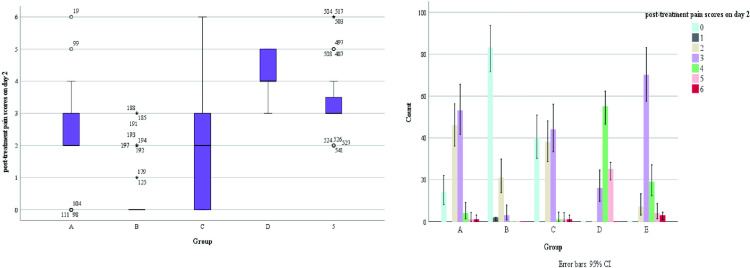
Post-treatment pain scores on day 2.

**Fig 11:**
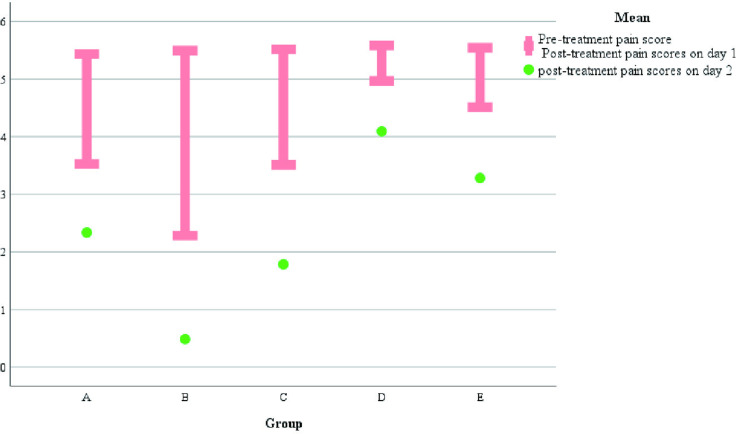
Comparison of pain scores before and after treatment.

**Fig 12:**
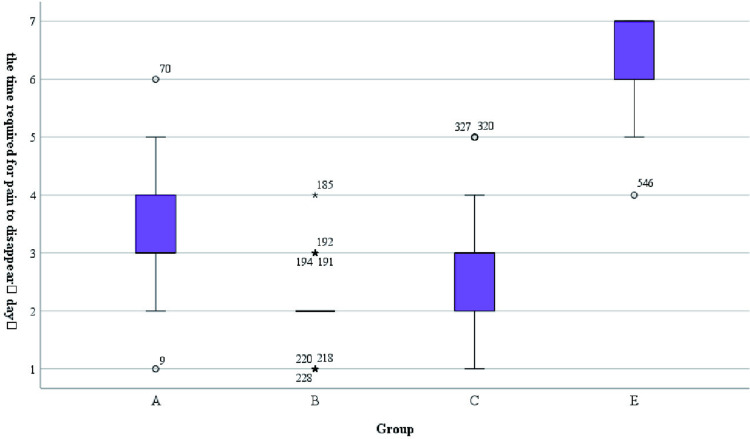
The time required for pain to disappear.

**Fig 13:**
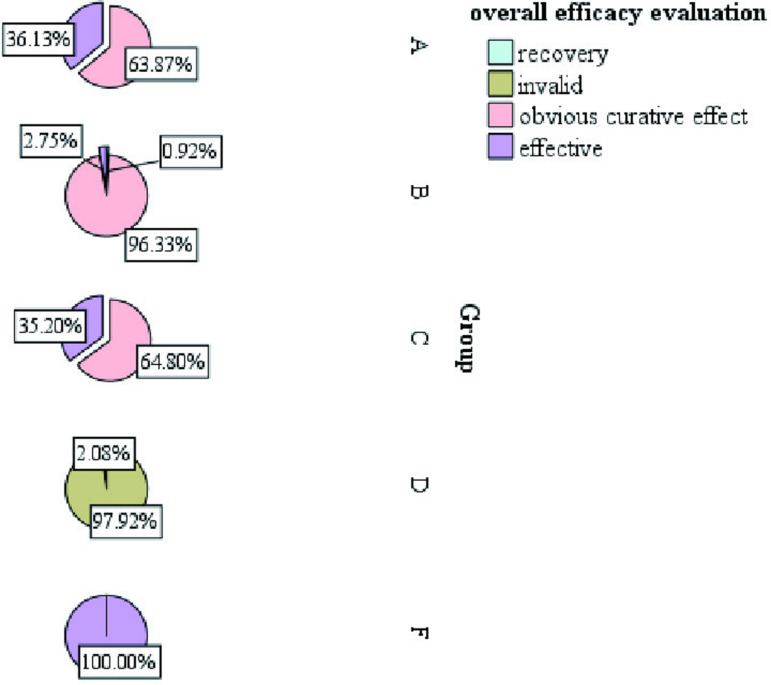
Overall efficacy evaluation.

It was observed that all patients who planned to undergo surgery were treated with surgery about one week of drug treatment (Fig 7).Some patients on outpatient oral drug therapy chose further surgery (68/103), and surgical data of some patients were missing, therefore, we chose to conduct the overall evaluation of drug efficacy after 1 week of drug treatment. We found a statistically significant difference in the number of days of medication prior to surgical treatment among patients in the 4 groups (A/B/C/D) (H = 265.292, p < 0.001), as well as fewer days of medication prior to surgical treatment among patients in group B compared to the other groups (Fig 7). There was no statistical significance in the occurrence of nipple discharge in the five groups (χ^2^ = 0.948, p = 0.918).Making box plots as well as histograms for non-normally distributed measures, we found no difference in pain scores before medication (H = 1.950, p = 0.745).Overall pain scores ranged from 4–7, with a predominance of 5–6 (Fig 8).The difference of pain scores on day 1 and day 2 after treatment was statistically significant (p < 0.001), and the pain symptoms were most significantly relieved in group B (Figs 9 and 10).Meanwhile, we compared the pain scores of patients in each group before and after treatment as a whole and found that patients in group B had significant pain relief after treatment (Fig 11).The difference in the time taken for the pain to disappear and the time taken to reduce the volume by half after treatment was statistically significant between the 5 groups of patients (p < 0.05).During treatment, it was found that the lesion volume in group D did not change significantly after treatment. Although the pain and symptoms were relieved after treatment, they were still accompanied on the course of the disease, while the complete relief of pain in group B took the shortest time (Fig 12).The difference in overall efficacy evaluation was statistically significant (H = 405.010, p = 0.000), with group B having the highest overall efficacy rate of 100% and group D having the lowest overall efficacy rate of 2.10%.In a two-by-two intergroup comparison, we found no statistically significant difference in the overall efficacy comparison between Group A and Group C (p = 0.909) (Table 4), this difference could also be detected in the efficacy profile pie chart (Fig 13).

**Table 3:** Evaluation of drug therapeutic effect.

Project/Group	A	B	C	D	E	F/H/χ2	p
case	119	109	125	96	103(68 cases were surgical patients)		
Number of days of preoperative medication,M(IQR)	5(1)	3 (1)	5 (1)	6 (1)	35 (21)	380.675	<0.001
Nipple discharge, n(%)						0.948	0.918
NO	51.00(42.86)	47.00(43.12)	59.00(47.20)	46.00(47.92)	47.00(45.63)		
Yes	68.00(57.14)	62.00(56.88)	66.00(52.80)	50.00(52.08)	56.00(54.37)		
Pre-treatment pain score,M(IQR)	5(1)	5(1)	6(1)	6(1)	6(1)	1.950	0.745
post-treatment pain scores on day 1,M(IQR)	3(1)	2(1)	3(1)	5(0)	5(1)	367.276	<0.001
post-treatment pain scores on day 2,M(IQR)	2(1)	0(0)	2(3)	4(1)	3(1)	327.724	<0.001
the time required for pain to disappear(day),M(IQR)	3(1)	2(1)	3(1)	*	7(1)	318.544	<0.001
the time required for the volume to be reduced by half(day),M(IQR)	4(2)	3(1)	4(2)	*	15(4)	303.854	<0.001
overall efficacy evaluation,n(%)						405.010	0.000
recovery	0(0.00)	1(0.92)	0(0.00)	0 (0.00)	0 (0.00)		
obvious curative effect	76(63.87)	105(96.33)	0(0.00)	0 (0.00)	0(0.00)		
Effective	43(36.13)	3(2.75)	81(64.80)	2 (2.08)	103(100.00)		
invalid	0(0.00)	0(0.00)	44(35.20)	94 (97.92)	0(0.00)		

**Table 4:** Pairwise comparisons of grouping variable.

Sample 1-Sample 2	Test Statistic	Std. Error	Std. Test Statistic	p
D-E	−141.49	20.787	−6.807	<0.001
D-A	287.422	20.102	14.298	0.000
D-C	289.558	19.885	14.561	0.000
D-B	364.907	20.509	17.792	0.000
E-A	145.933	19.72	7.4	<0.001
E-C	148.068	19.499	7.593	<0.001
E-B	223.417	20.136	11.096	0.000
A-C	−2.135	18.767	−0.114	0.909
A-B	−77.485	19.427	−3.988	0.000
C-B	75.349	19.203	3.924	0.000

### 2.4 Evaluation of the safety of drug therapy (Table 5)

During drug therapy, patients were closely monitored for possible side effects, and we found that none of the patients in the five groups experienced side effects such as abnormalities in liver and kidney function, water-electrolyte disorders, or peptic ulcers during drug therapy. There was no statistically significant difference between the five groups in the occurrence of side effects such as rash, diarrhoea and hyperglycaemia (p > 0.05); in the occurrence of nausea (vomiting) and skin pigmentation deepening side effects, Group E was higher than the other four groups, while the other four groups did not have such side effects, and at the same time, the difference was statistically significant (p < 0.001).In terms of weight gain (full moon face), nervous excitability (insomnia), and menstrual disorders, group E was higher than the other 4 four patients, and except for group D, who was not treated with hormones, the patients in group B were lower than the other 3 groups, while the difference was statistically significant (p < 0.001).

**Table 5:** Evaluation of the safety of drug therapy (n(%)).

Project/Group	A	B	C	D	E	χ2	p
nausea (vomiting)						39.883	<0.001
NO	119(100.00)	109(100.00)	125(100.00)	96(100.00)	94(91.30)		
YES	0(0.00)	0(0.00)	0(0.00)	0(0.00)	9(8.70)		
rash						8.750	0.067
NO	119(100.00)	109(100.00)	125(100.00)	96(100.00)	101(98.10)		
YES	0(0.00)	0(0.00)	0(0.00)	0(0.00)	2(1.90)		
diarrhoea						8.750	0.067
NO	119(100.00)	109(100.00)	125(100.00)	96(100.00)	101(98.10)		
YES	0(0.00)	0(0.00)	0(0.00)	0(0.00)	2(1.90)		
peptic ulcers						*	*
NO	119(100.00)	109(100.00)	125(100.00)	96(100.00)	103(100.00)		
YES	0(0.00)	0(0.00)	0(0.00)	0(0.00)	0(0.00)		
water-electrolyte disorders						*	*
NO	119(100.00)	109(100.00)	125(100.00)	96(100.00)	103(100.00)		
YES	0(0.00)	0(0.00)	0(0.00)	0(0.00)	0(0.00)		
hyperglycaemia						5.398	0.248
NO	112(94.10)	104(95.40)	120(96.00)	96(100.00)	99(96.10)		
YES	7(5.90)	5(4.60)	5(4.00)	0	4(3.90)		
abnormalities in liver and kidney function						*	*
NO	119(100.00)	109(100.00)	125(100.00)	96(100.00)	103(100.00)		
YES	0(0.00)	0(0.00)	0(0.00)	0(0.00)	0(0.00)		
weight gain (full moon face)						174.132	<0.001
NO	112(94.10)	108(99.10)	117(93.60)	96(100.00)	51(49.50)		
YES	7(5.90)	1(0.90)	8(6.40)	0	52(50.50)		
menstrual disorders						174.489	<0.001
NO	117(98.30)	109(100.00)	123(98.40)	95(99.00)	60(58.30)		
YES	2(1.70)	0	2(1.60)	1(1.00)	43(41.70)		
skin pigmentation						234.410	<0.001
NO	119(100.00)	109(100.00)	125(100.00)	96(100.00)	54(52.40)		
YES	0(0.00)	0(0.00)	0(0.00)	0(0.00)	49(47.60)		
nervous excitability (insomnia)						115.714	<0.001
NO	115(96.60)	109(100.00)	119(95.20)	95(99.00)	67(65.00)		
YES	4(3.40)	0(0.00)	6(4.80)	1(1.00)	36(35.00)		

### 2.5 Prognosis evaluation and follow-up results (Table 6)

The patients included in the study were followed for 1 year. In terms of contralateral new occurrence, there was no statistically significant difference among the 5 groups (p > 0.05); in terms of relapse, the relapse rate of group B was lower than that of the other 4 groups, and the relapse rate of group D was the highest, the difference was statistically significant (p < 0.001). A satisfaction survey was also conducted and it was found that group B had the highest satisfaction rate and group E had the lowest satisfaction rate and the difference was statistically significant (p = 0.041).

**Table 6:** Prognosis evaluation of drug therapy.

Project/Group	A	B	C	D	E	χ2	p
case	119	109	125	96	103		
relapse, n(%)						12.505	0.014
NO	117(98.30)	108(99.10)	123(98.40)	88(91.70)	99(96.10)		
YES	2(1.70)	1(0.90)	2(1.60)	8(8.30)	4(3.90)		
new occurrence, n(%)						2.256	0.689
NO	116(97.50)	108(99.10)	122(97.60)	92(95.80)	100(97.10)		
YES	3(2.50)	1(0.90)	3(2.40)	4(4.20)	3(2.90)		
satisfaction, n(%)						9.973	0.041
unsatisfied	5(4.20)	1(0.90)	5(4.00)	7(7.30)	10(9.70)		
satisfied	114(95.80)	108(99.10)	120(96.00)	89(92.70)	93(90.30)		

### 2.6 According to different pathological types, the difference in efficacy between the groups was compared

Dexamethasone was included in the treatment of patients in groups A, B and C. A total of 353 patients in groups A, B and C included in the study were divided into GLM and PCM groups according to the different pathological types, and the efficacy of the drug treatment was evaluated by comparing the drug treatment between the groups. We found no statistically significant differences in the number of days of medication before surgical treatment, pre-treatment pain scores, post-treatment pain scores, time taken for pain to disappear, and the number of days taken to halve the volume after treatment (p > 0.05). As for the overall efficacy evaluation, GLM patients had a higher rate of apparent efficacy as well as healing than PCM patients, and the difference was statistically significant (H = 8.359, p = 0.015) (Table 7).

**Table 7:** Evaluation of therapeutic effect of different pathological types.

Project/Group	PCM	GLM	F/H/χ2	p
case	148	205		
Days of medication before surgery,M(IQR)	5(1)	5(1)	0.007	0.932
Pre-treatment pain score,M(IQR)	5(1)	6(1)	0.367	0.544
post-treatment pain scores on day 1,M(IQR)	3(1)	3(2)	2.721	0.099
post-treatment pain scores on day 2,M(IQR)	2(3)	2(3)	2.621	0.105
the time required for pain to disappear(day),M(IQR)	3(1)	3(1)	0.278	0.597
the time required for the volume to be reduced by half(day),M(IQR)	3(1)	4(1)	2.352	0.125
overall efficacy evaluation,n(%)			8.359	0.015
recovery	0(0.00)	1(0.50)		
obvious curative effect	99(66.90)	163(79.50)		
Effective	49(33.10)	41(20.00)		
invalid	0(0.00)	0(0.00)		

### 2.7 Analysis of multiple factors affecting satisfaction outcome and drawing of forest map

**Fig 14:**
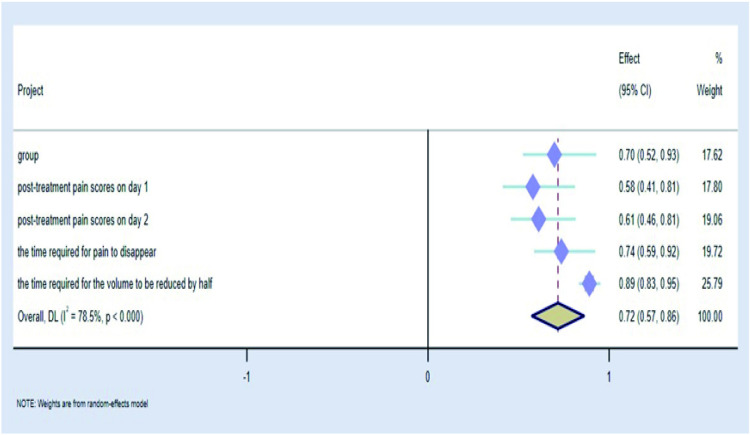
Forest diagrams for efficacy evaluation.

**Fig 15:**
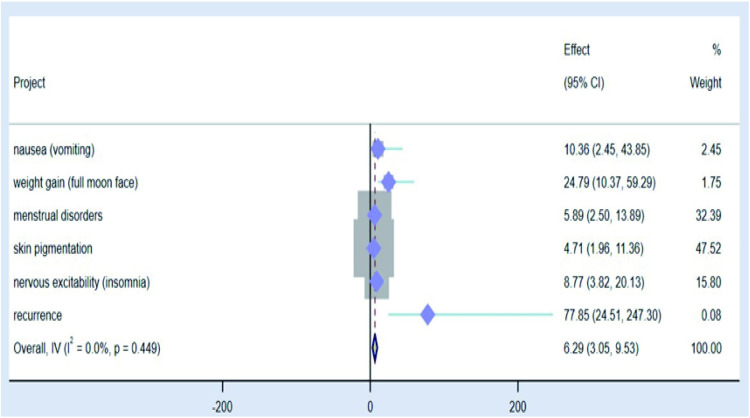
Forest diagrams of drug safety and prognostic indicators.

**Fig 16:**
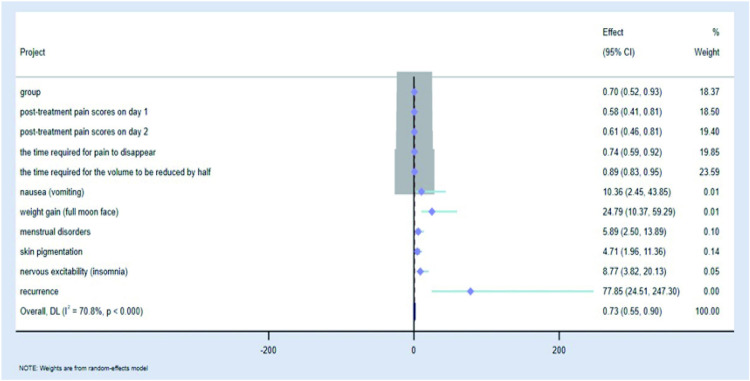
Forest diagrams of all factors.

We used the post-treatment satisfaction outcome as a dependent variable and assigned values to the satisfactory outcome, with values of 0 = unsatisfied and 1 = satisfied, and logistic regression analyses were performed on the statistically significant values in the study to analyse the risk factors affecting the treatment satisfaction outcome. Through this retrospective study, we found that the relevant factors affecting treatment satisfaction included the grouping of treatment regimens, pain scores on days 1 and 2 post-treatment, the time required for pain to disappear, the time required for the volume to be reduced by half, the associated side-effects, and recurrence (Table 8). Forest plots of risk factors affecting satisfaction outcomes in logistic regression analysis were analysed using STATA 15 software.I^2^ test was used to evaluate statistical heterogeneity. When p > 0.1 and I^2^ < 50%, the study was determined to be homogeneous and analyzed using a fixed effect model. When p < 0.1 and I^2^ > 50%, the study was identified as heterogeneity and a random effect model was used. Forest diagrams of relevant efficacy evaluation indicators(Figs 14 and 16): the grouping of treatment regimens, pain scores on days 1 and 2 post-treatment, the time required for pain to disappear, the time required for the volume to be reduced by half significantly affect satisfaction. As the risk factors affecting satisfaction, the number of days required for the volume to be reduced by half after treatment had a significant impact, and the OR (odds ratio) value of influencing satisfaction was 0.891. 95%CI was 0.833–0.952, accounting for the highest weight (25.79%). Forest diagrams of drug safety and prognostic indicators: Associated side effects occurring during treatment also affect satisfaction, with menstrual disorders as well as skin pigmentation deepening carrying higher weights of 32.39% and 47.52%, respectively. (Figs 15 and 16). (The grouping here referred to the grouping according to the medication regimen).

**Table 8:** Logistic regression analysis affecting satisfaction outcome.

Project	Regression coefficient(B)	Wald	Exp(B)(95%CI)	p
group	0.359	6.066	0.698(0.524-0.929)	0.014
Onset time	0.011	1.269	1.011(0.992-1.030)	0.260
pathological diagnosis	0.088	0.049	1.092(0.501-2.379)	0.825
Number of days of preoperative medication	0.023	2.964	0.978(0.953-1.003)	0.085
post-treatment pain scores on day 1	0.546	10.063	0.579(0.413-0.812)	0.002
post-treatment pain scores on day 2	0.493	11.207	0.611(0.457-0.815)	<0.001
the time required for pain to disappear	0.307	7.021	0.736(0.586-0.923)	0.008
the time required for the volume to be reduced by half	0.012	11.465	0.891(0.833-0.952)	<0.001
overall efficacy evaluation	0.515	1.301	0.597(0.246-1.448)	0.254
nausea (vomiting)	2.338	10.086	10.360(2.448-43.851)	0.001
weight gain (full moon face)	3.211	52.075	24.792(10.366-59.293)	<0.001
menstrual disorders	1.773	16.427	5.891(2.499-13.887)	<0.001
skin pigmentation	1.550	11.928	4.712(1.955-11.357)	<0.001
nervous excitability (insomnia)	2.171	26.260	8.771(3.823-20.126)	<0.001
recurrence	4.355	54.532	77.850(24.507-247.299)	<0.001

## 3 Discussion

NPM is a type of benign breast disease that accounts for approximately 4–5% of benign breast diseases [18], and the prevalence of NPM has increased significantly in recent years [19].The etiology of NPM is largely unknown [20], as there is a lack of large-scale epidemiological investigations related to NPM, as well as relatively little clinical or basic research on NPM [21], thus posing great challenges and difficulties in treatment for both physicians and patients [22]. However, this disease is extremely similar to breast cancer in terms of imaging characteristics [23], there are easy to miss the diagnosis, misdiagnosis or delayed diagnosis, etc., which belongs to the difficult breast diseases. Some patients are prone to repeated illness and breast deformity, which seriously affects the physical and mental health of patients.

In exploring the treatment of NPM, we continue to summarize our experience and find the best treatment options for patients with NPM. As a specialized hospital for women and children, it has its own dominant disease. In this retrospective study, we made use of the advantage that NPM is the most common disease in our department, and summarized the advantageous empirical drugs in clinical practice, trying to find out the optimal treatment plan. In the current treatment of NPM common medications include glucocorticoids, usually prednisone and methylprednisolone tablets are commonly used.

Dexamethasone(DEX), a long-acting glucocorticoid, has stronger anti-inflammatory, anti-allergic and anti-toxic effects than prednisone (medium acting glucocorticoid), and is more effective than natural glucocorticoids [15].DEX can reduce the exudation, vasodilation and phagocytosis of inflammatory cells in the early stage of inflammation, and inhibit fibrosis by reducing the proliferation of fibroblasts when inflammation is severe [24]. DEX has been widely used in the treatment of various diseases, including autoimmune diseases, allergies and cancer, etc. In the course of treatment, DEX is also commonly used in our department as the basic drug for NPM, which is administered intravenously for a short period of time, but different doctors have chosen different treatment protocols based on their clinical experience. In this retrospective study, we found that there were five different groups of treatment plans, and the age of patients included in this retrospective study was mainly between 30 and 40 years old, which was consistent with the high-risk age group reported in previous studies [4].The BMI values of patients with different pathological subtypes were mainly concentrated in the range of 20–30 Kg/m², and previous studies have found that overweight (obesity) or late menarche is an independent risk factor for PDM [5], and there was no difference in the baseline values of the patients in the present study between the groups (p > 0.05), including the BIM values and the age of menarche, which ensured the homogeneity of the baseline values of the present study. However, the time of onset was statistically significant between the 5 groups of patients (p = 0.012), and a careful search for the cause revealed that the time of onset of patients in group E was significantly more than that of the other 4 groups, which may be related to the delay in treatment due to insufficient knowledge of the disease, the patient’s non-cooperation with the treatment, or the choice of the treatment plan by the first doctor.

This retrospective study mainly adopted breast color ultrasound as an evaluation tool, because it was found that breast ultrasound, as the best imaging method for patients suspected of NPM, could be used to evaluate the characteristics of the lesions and the scope of the lesions [25].In this retrospective study, we found that most NPM patients underwent surgical treatment about 1 week after drug treatment, so we chose to conduct the overall evaluation of drug efficacy 1 week after drug treatment. It was found that the number of days of drug treatment before surgery in group B was less than that in other groups (p < 0.001), thus shortening the time of preoperative treatment. Most patients with NPM have a combination of symptoms such as pain and discomfort in the affected breast. We performed pain scores on the patients included in the study and found that there was a difference in pain scores on the first day as well as the second day after treatment among the five groups, and that the patients in group B had the most significant relief of pain symptoms (p < 0.001).And the pain symptoms in group B were most significantly relieved (p < 0.001). Meanwhile, we compared the pain scores of patients in each group before and after treatment as a whole and found that patients in Group B had significant pain relief after treatment. The time taken for the pain to disappear and the time taken to reduce the volume by half after treatment was statistically significant between the five groups of patients (p < 0.05), and in the treatment we found that there was no significant change in the volume of the lesion after treatment in group D, which was still accompanied by the course of the disease despite the reduction of the pain and the relief of the symptoms after the treatment, and that group B took the shortest time for the pain to completely subside. From the treatment, we could find that dexamethasone and metronidazole/levofloxacin treatment combination had better benefits for NPM patients, and some related consensus and studies [7] also proposed that lipophilic antibiotics in the treatment of GLM may have higher benefits [26].The difference in overall efficacy evaluation was statistically significant (p < 0.001), and the overall effective rate of group B was the highest (100%).The overall effective rate of group D was the lowest (2.10%).In the comparison between the two groups, we found that there was no statistical significance in the comparison of overall efficacy between group A and group C (p > 0.05). Although group C had one more group of cephalosporin antibiotics than group A, the therapeutic effect was similar to that of group A. Therefore, adding cephalosporin antibiotics to the treatment of NPM may not bring additional benefits to NPM. In contrast, no treatment with dexamethasone was used in group D, which had the poorest efficacy in the treatment of NPM. It could be seen that the use of antibiotics alone in the treatment of NPM may not be effective in treating the disease.

This retrospective study found that the efficacy of dexamethasone in the treatment of NPM was significant, but we also paid attention to the possible side effects of treatment in the treatment. Madamsetty [24] found that high dose application of dexamethasone may cause various side effects such as hypertension, adrenal suppression, osteoporosis and others through his study. Therefore, during the drug treatment, we closely monitored the possible side effects of the patients, and we found that during the drug treatment, none of the patients in the five groups had side effects such as abnormalities in liver and kidney functions, water-electrolyte disorders, and peptic ulcers. There was no statistical significance in the occurrence of side effects such as rash, diarrhea and hyperglycemia among the 5 groups (p > 0.05). The side effects of nausea (vomiting) and skin pigmentation in group E were higher than those in the other 4 groups, but no such side effects occurred in the other 4 groups, and the difference was statistically significant (p < 0.001).In terms of weight gain (full moon face), nervous excitability (insomnia), menstrual disorders, group E was higher than the other 4 groups of patients, and group B patients were lower than the other 3 groups, excluding group D, who were not treated with hormones, while the difference was statistically significant (p < 0.001).Analysing the reasons for this quadrant, it is possible that the best efficacy of the treatment regimen in group B, and thus the shorter duration of dosing, and therefore the lower chances of drug side-effects, makes it safe and reliable for short-term dexamethasone application in the treatment of NPM.

During the 1-year follow-up of the patients included in the study, we found that contralateral new lesions appeared in very few patients, but the difference was not statistically significant (p > 0.05).It was also found that relapse occurred in the ipsilateral breast after surgery, and the relapse rate of patients in group B was lower than that of the other four groups, and group D had the highest relapse rate, with a statistically significant difference (p < 0.001).For the study, we can find that dexamethasone combined with levofloxacin/metronidazole can improve the prognosis of the ipsilateral breast in the treatment of NPM, but the specific mechanism needs to be further explored. A satisfaction survey was also conducted and it was found that group B had the highest satisfaction rate and group E had the lowest satisfaction rate and the difference was statistically significant (p < 0.05). When 353 patients with dexamethasone included in therapeutic drugs were grouped according to different pathologic types, no difference was found among the relevant groups (p > 0.05), while the overall efficacy evaluation found that GLM patients had a higher efficacy and recovery rate than PCM patients (p < 0.05). The study also found that the number of days required for the volume to be reduced by half after treatment was the most influential factor in the satisfaction survey, with the greatest weight.

To sum up, this study has many strengths: (1) As a large maternal and child specialized hospital, it has a perfect database of scientific research data platform, meanwhile, NPM is a common disease in our department, with a large number of samples, and our department has a perfect NPM treatment as well as a follow-up system, which guarantees the maximum completeness of the information in this study and also provides reliable clinical information for the results of this study. (2) The current study utilizes different NPM treatment protocols to comprehensively evaluate the role of DEX in the treatment, which can provide a strong clinical reference value for the current treatment of NPM as well as the direction of treatment. It also can provide a strong clinical evidence for the development and update of the NPM treatment guidelines.(3) To date, this study is the first to examine the outstanding efficacy and benefits of DEX in combination with levofloxacin/metronidazole in the treatment of NPM, and the findings of this study provide a superior choice of clinical therapeutic regimen in the treatment of NPM, and these findings bring a new light to patients with NPM. However, there are still some limitations in this retrospective study: (1) DEX was administered by a single route in this study. We chose intravenous dexamethasone treatment and did not add oral dexamethasone treatment for comparison, so the comparison of the efficacy of different routes of dexamethasone administration in the treatment of NPM could not be performed at the same time. (2)There is a lack of prospective studies of DEX in the treatment of NPM, including the minimum effective dose of DEX and the optimal course of treatment, and later studies on different doses and courses of DEX can be increased to evaluate the role of DEX in the treatment of NPM. (3) The current study is a single-center study and lack a multicenter efficacy comparison. The sample size is increased at a later stage, while the clinical efficacy of different doses and routes of administration of DEX in the treatment of NPM is evaluated in a multicenter and prospective manner. (4) Unknown mechanism of action: The mechanism of action of DEX combined with levofloxacin/metronidazole in the treatment of NPM is currently unclear, and studies of the relevant mechanism of action will be conducted at a later stage.

## 4 Conclusion

Dexamethasone has many advantages when combined with levofloxacin/metronidazole in the treatment of NPM. Primarily, it can significantly alleviate the pain symptoms caused by the disease and effectively reduce the size of the lesion. At the same time, for patients who plan to undergo surgery, it can significantly reduce the number of days of preoperative drug treatment, and the overall effective rate is the highest. Secondly, it has less side reaction and high safety when applied for a short time. At the same time, the risk of relapse of the ipsilateral breast was less and the satisfaction of the patients was higher. When treated medically, GLM patients had higher overall efficacy rates as well as higher cure rates than PCM patients. Dexamethasone in combination with metronidazole/levofloxacin can be chosen as a clinical treatment option for NPM patients, especially for GLM patients. This provides a strong basis for the formulation of clinical treatment guidelines for NPM.

## Supporting information

S1 Data The data about the treatment of non⁃puerperal mastitis. (XLSX)
